# Fertility history and intentions of married women, China 

**DOI:** 10.2471/BLT.23.289736

**Published:** 2024-01-31

**Authors:** Qin Li, Rui Yang, Zehong Zhou, Weiping Qian, Jian Zhang, Ze Wu, Lei Jin, Xueqing Wu, Cuilian Zhang, Beihong Zheng, Jichun Tan, Guimin Hao, Shangwei Li, Yongxiu Hao, Danni Zheng, Yuanyuan Wang, Rong Li, Ping Liu, Jie Qiao

**Affiliations:** a State Key Laboratory of Female Fertility Promotion, Center for Reproductive Medicine, Department of Obstetrics and Gynaecology, Peking University Third Hospital, 49 North Garden Road, Beijing, 100191, China.; b Guangzhou Institute of Paediatrics, Guangzhou Medical University, Guangzhou, China.; c Department of Reproductive Medicine, Peking University Shenzhen Hospital, Shenzhen, China.; d Key Laboratory of Cell Differentiation and Apoptosis of Chinese Ministry of Education, Shanghai Jiao Tong University, Shanghai, China.; e Department of Reproductive Medicine, The First People's Hospital of Yunnan Province, Kunming, China.; f Reproductive Medicine Center, Huazhong University of Science and Technology, Wuhan, China.; g Children's Hospital of Shanxi and Women Health Center of Shanxi, Affiliated Hospital of Shanxi Medical University, Taiyuan, China.; h Reproductive Medical Center, Henan Provincial People's Hospital, Zhengzhou, China.; i Reproductive Medicine Center, Fujian Provincial Maternity and Children's Hospital, Fuzhou, China.; j Center of Reproductive Medicine, Shengjing Hospital of China Medical University, Shenyang, China.; k Department of Reproductive Medicine, The Second Hospital of Hebei Medical University, Shijiazhuang, China.; l Division of Reproductive Medicine, West China Second University Hospital of Sichuan University, Chengdu, China.; m National Clinical Research Center for Obstetrics and Gynaecology, Peking University Third Hospital, Beijing, China.; n Key Laboratory of Assisted Reproduction (Peking University), Ministry of Education, Beijing, China.; o Beijing Key Laboratory of Reproductive Endocrinology and Assisted Reproductive Technology, Beijing, China.

## Abstract

**Objective:**

To estimate the proportion of married women in China who intend to become pregnant given the country’s pronatalist population policy and to investigate fecundity, with an emphasis on the influence of socioeconomic factors.

**Methods:**

A nationally representative survey of 12 815 married women aged 20 to 49 years (mean: 36.8 years) was conducted during 2019 and 2020. All completed questionnaires, 10 115 gave blood samples and 11 710 underwent pelvic ultrasound examination. Fertility intention was the desire or intent to become pregnant combined with engagement in unprotected sexual intercourse. We defined infertility as the failure to achieve pregnancy after 12 months or more of unprotected intercourse. We considered an anti-Müllerian hormone level < 1.1 ng/mL and an antral follicular count < 7 as indicating an abnormal ovarian reserve.

**Findings:**

Fertility intentions were reported by 11.9% of women overall but by only 6.1% of current mothers (weighted percentages). Fertility intention was significantly less likely among women in metropolises (odds ratio, OR: 0.38; 95% confidence interval, CI: 0.31–0.45) and those with a higher educational level (OR: 0.74; 95% CI: 0.62–0.88). Overall, 18.0% had experienced infertility at any time and almost 30% had an abnormal ovarian reserve on assessment. An abnormal ovarian reserve and infertility were less likely in women in metropolises (*P* < 0.05) but more likely in obese women (*P* < 0.05).

**Conclusion:**

The willingness of Chinese married women to give birth remained low, even with relaxation of the one-child policy.

## Résumé

**Objectif:**

Déterminer le pourcentage de femmes mariées prévoyant de tomber enceintes en Chine compte tenu de la politique démographique pronataliste du pays, et étudier la fécondité à la lumière des facteurs socioéconomiques.

**Méthodes:**

Une enquête représentative à l'échelle nationale portant sur 12 815 femmes mariées âgées de 20 à 49 ans (moyenne: 36,8 ans) a été organisée entre 2019 et 2020. Toutes les participantes ont rempli des questionnaires, 10 115 d'entre elles ont fourni des échantillons de sang et 11 710 ont subi une échographie pelvienne. L'intention de grossesse correspondait au désir ou à la volonté de tomber enceinte, combinée à des rapports sexuels non protégés. Nous avons défini l'infertilité comme l'incapacité à concevoir un enfant après minimum 12 mois de rapports sexuels non protégés. Enfin, nous avons considéré qu'un taux d'hormones antimüllérienne < 1,1 ng/mL et un compte folliculaire antral < 7 indiquaient une réserve ovarienne anormale.

**Résultats:**

Au total, 11,9% des femmes ont exprimé leur désir de grossesse, mais seulement 6,1% de celles étant déjà mères (pourcentages pondérés). Cette intention était nettement moins marquée chez les femmes résidant en métropole (odds ratio, OR: 0,38; intervalle de confiance de 95%, IC: 0,31–0,45) et chez celles affichant un niveau d'éducation plus élevé (OR: 0,74; IC de 95%: 0,62-0,88). Globalement, 18,0% ont connu l'infertilité à un moment donné et 30% présentaient une réserve ovarienne anormale lors de l'examen. Les phénomènes de réserve ovarienne anormale et d'infertilité étaient moins susceptibles de se manifester chez les femmes résidant en métropole (*P* < 0,05) mais plus susceptibles chez les femmes obèses (*P* < 0,05).

**Conclusion:**

En Chine, rares sont les femmes mariées à vouloir procréer, même avec l'assouplissement de la politique de l'enfant unique.

## Resumen

**Objetivo:**

Estimar el porcentaje de mujeres casadas en China que tienen intención de quedarse embarazadas dada la política demográfica pronatalista del país e investigar la fecundidad, haciendo énfasis en la influencia de los factores socioeconómicos.

**Métodos:**

Durante 2019 y 2020, se realizó una encuesta representativa a nivel nacional de 12 815 mujeres casadas de entre 20 y 49 años (media: 36,8 años). Todas las participantes completaron cuestionarios, 10 115 aportaron muestras de sangre y 11 710 se sometieron a un examen de ultrasonido pélvico. La intención de fertilidad fue el deseo o la intención de quedar embarazada combinada con la participación en relaciones sexuales sin protección. Se definió la infertilidad como la no consecución de un embarazo tras 12 meses o más de relaciones sexuales sin protección. Asimismo, se consideró que un nivel de hormona antimülleriana <1,1 ng/mL y un recuento folicular antral <7 indicaban una reserva ovárica anormal.

**Resultados:**

El 11,9% de las mujeres informó de su intención de fertilidad, pero solo el 6,1% de las madres actuales (porcentajes ponderados). La intención de fertilidad era significativamente menos probable entre las mujeres de las metrópolis (razón de posibilidades, OR: 0,38; intervalo de confianza del 95%, IC: 0,31-0,45) y las que tenían un nivel educativo más alto (OR: 0,74; IC del 95%: 0,62-0,88). En general, el 18,0% había experimentado infertilidad en algún momento y casi el 30% tenía una reserva ovárica anormal en la evaluación. La reserva ovárica anormal y la infertilidad eran menos probables en las mujeres de las metrópolis (*p* <0,05), pero más probables en las mujeres obesas (*p* <0,05).

**Conclusión:**

La disposición de las mujeres chinas casadas a dar a luz sigue siendo baja, incluso con la flexibilización de la política de un solo hijo.

## ملخص

**الغرض:**

تقدير نسبة السيدات المتزوجات في الصين اللاتي ينوين الحمل في ظل السياسة السكانية المشجعة على الإنجاب في الدولة، فضلًا عن دراسة الكفاءة التناسلية، مع التركيز على تأثير العوامل الاجتماعية والاقتصادية.

**الطريقة:**

تم إجراء مسح تمثيلي على المستوى الوطني لعدد 12815 من السيدات المتزوجات، تتراوح أعمارهن بين 20 و49 عامًا (المتوسط: 36.8 عامًا) خلال عامي 2019 و2020. أكملت جميع المشاركات الاستبيانات، وأعطت 10115 منهن عينات للدم، وخضع 11710 منهم لفحص الحوض بالموجات فوق الصوتية. نية الخصوبة هي الرغبة أو النية في الحمل، والمرتبطة بالانخراط في اتصال جنسي غير محمي. قمنا بتعريف العقم بأنه الفشل في تحقيق الحمل بعد 12 شهرًا، أو أكثر، من الاتصال الجنسي غير المحمي. لقد اعتبرنا كل من مستوى الهرمون المضاد لمولر بنسبة أقل من 1.1 نانوغرام/مل، وعدد الحويصلات الجيبية الأقل من 7، بمثابة مؤشر على احتياطي غير طبيعي للمبيض.

**النتائج:**

تم الإبلاغ عن نوايا الخصوبة بنسبة %11.9 من السيدات بشكل عام، ولكن بنسبة %6.1 فقط من الأمهات الحاليات (النسب المئوية المرجحة). كانت نية الخصوبة أقل احتمالًا بشكل ملحوظ بين السيدات في المدن الكبرى (نسبة الاحتمالات (OR): 0.38؛ بفاصل ثقة مقداره %95: 0.31 إلى 0.45) وبين السيدات ذوات المستوى التعليمي الأعلى (نسبة الاحتمالات: 0.74؛ ‏بفاصل ثقة مقداره %95: 0.62 إلى 0.88). بشكل عام، كانت %18.0 منهن قد عانين من العقم في أي وقت، وكان لدى %30 منهن تقريبًا احتياطي غير طبيعي للمبيض عند التقييم. كان الاحتياطي غير الطبيعي للمبيض والعقم أقل احتمالا لدى السيدات في المدن الكبرى (نسبة الاحتمال أقل من 0.05)، ولكن أكثر احتمالا عند السيدات البدينات (نسبة الاحتمال أقل من 0.05).

**الاستنتاج:**

ظلت رغبة السيدات الصينيات المتزوجات في الإنجاب منخفضة، حتى مع تخفيف سياسة الطفل الواحد.

## 摘要

**目的:**

预估在中国鼓励生育人口政策下已婚女性有意愿怀孕的比例，并调查生育能力情况，同时重点关注社会经济因素的影响。

**方法:**

在 2019 年和 2020 年期间，对 12,815 名年龄在 20 至 49 岁之间的已婚女性（平均年龄：36.8 岁）进行了一项具有全国代表性的调查。所有参与者都完成了问卷调查，其中 10,115 人提供了血液样本，11,710 人接受了盆腔超声检查。生育意图是指通过未采取避孕措施的性交怀孕的愿望或意愿。我们将不孕症定义为在 12 个月或更长的时间内进行未采取避孕措施的性交后仍未能怀孕的情况。我们认为抗苗勒管激素水平低于 1.1 ng/mL，窦卵泡计数低于 7 即表示卵巢储备功能异常。

**结果:**

总体而言，报告显示 11.9% 的女性有生育意愿，但只有 6.1% 的已为人母的女性有生育意愿（加权百分比）。大都市女性（比值比，OR：0.38；95% 置信区间，CI：0.31-0.45）和受教育程度较高的女性（OR：0.74；95% CI：0.62-0.88）的生育意愿明显偏低。总体而言，18.0% 的女性曾有过不孕经历，近 30% 的女性在评估时其卵巢储备功能异常。卵巢储备功能异常和不孕症在大都市女性中发生率较低 (*P* < 0.05)，而在肥胖女性中发生率较高 (*P* < 0.05)。

**结论:**

即使中国独生子女政策已有所放宽，已婚女性的生育意愿仍然很低。

## Резюме

**Цель:**

Оценить долю замужних женщин в Китае, которые собираются забеременеть, с учетом пронаталистской демографической политики страны и исследовать уровень фертильности с акцентом на влияние социально-экономических факторов.

**Методы:**

В 2019 и 2020 годах был проведен репрезентативный опрос на национальном уровне среди 12 815 замужних женщин в возрасте от 20 до 49 лет (средний возраст: 36,8 года). Все участницы заполнили анкеты. 10 115 женщин сдали кровь на анализ, 11 710 прошли ультразвуковое исследование органов малого таза. Под намерением завести ребенка понималось желание или намерение забеременеть в сочетании с незащищенным половым актом. Под бесплодием подразумевается отсутствие беременности после 12 месяцев и более незащищенных половых контактов. Считалось, что уровень антимюллерова гормона менее 1,1 нг/мл и количество антральных фолликулов менее 7 свидетельствуют о снижении овариального резерва.

**Результаты:**

О намерениях завести ребенка сообщили в целом 11,9% женщин, но только 6,1% женщин являются матерями в настоящее время (массовая доля). Намерение завести ребенка было значительно менее вероятным среди жительниц мегаполисов (отношение шансов, ОШ: 0,38; 95%-й доверительный интервал, ДИ: 0,31–0,45) и среди женщин с более высоким уровнем образования (ОШ: 0,74; 95%-й ДИ: 0,62–0,88). В общей сложности у 18,0% женщин в любое время наблюдалось бесплодие, почти у 30% выявлено снижение овариального резерва. Снижение овариального резерва и бесплодие были менее вероятны у жительниц мегаполисов (*P* < 0,05), но более вероятны у женщин с ожирением (*P* < 0,05).

**Вывод:**

Готовность замужних женщин Китая рожать детей остается низкой, даже после смягчения политики одного ребенка.

## Introduction

Fertility rates have been on the decline globally, with recent reports predicting that 23 countries will observe the halving of their population by the end of the century.^1^ Although a smaller population may be advantageous for the environment, an aging population presents other challenges to socioeconomic well-being.^2^ A key reason for the decline in fertility rates could be people prioritizing their education and careers, combined with the wide availability of contraception.^3^^,^^4^ However, poor fecundity could be equally responsible for the low birth rate.

Previous studies indicate that an increasing number of couples encounter challenges in achieving pregnancy.^5^^,^^6^ Infertility has been estimated to affect 8% to 12% of reproductive-aged couples worldwide.^7^ In light of the trend towards delayed parenthood, fertility tests are becoming more important in reproductive counselling, particularly when recommending fertility treatment. However, most existing evidence on infertility has been derived solely from questionnaire-based surveys, from which it is difficult to draw clear conclusions about the fecundity of women of reproductive age in general.

Globally, a large percentage of infertile women live in China – one of the most densely populated countries.^8^ However, infertility has long been overlooked due to the growing size of the Chinese population and the country’s family planning policy.^9^ In 2013, China started to relax its one-child policy to help cope with an aging population and to counter very low fertility. Despite the government enacting a series of policies that targeted married women of childbearing age who had had a previous delivery,^10^ the total fertility rate in the country dropped to 1.3 children per woman in 2020.^11^ In addition, the total fertility rate fell further to 1.15 children per woman in 2021 because of the impact of the coronavirus disease 2019 (COVID-19) pandemic. In this context, detailed data on fertility intention and fecundity in the general population are essential for developing evidence-based policies. 

The aim of our study was to bridge the evidential gap on fertility intention and fecundity in China by analysing data from the last iteration of the China fertility survey of married women, which investigated fertility behaviour and collected information on laboratory and ultrasound markers of fecundity. In particular, we estimated the proportion of married Chinese women who wished to become pregnant in the context of the country’s more relaxed population policy, studied their fecundity (i.e. infertility and ovarian reserve), and assessed how pregnancy desire and fecundity were influenced by sociodemographic factors. In addition, we intended to provide baseline data on fertility intentions before the onset of the COVID-19 pandemic.

## Methods

The 2020 China fertility survey of married women was one of a series of surveys that has been conducted since 2005 with the aim of investigating fertility and factors influencing fertility among couples in the general Chinese population.^12^^–^^14^ The 2020 survey took place between January 2019 and December 2020.

**Fig. 1:**
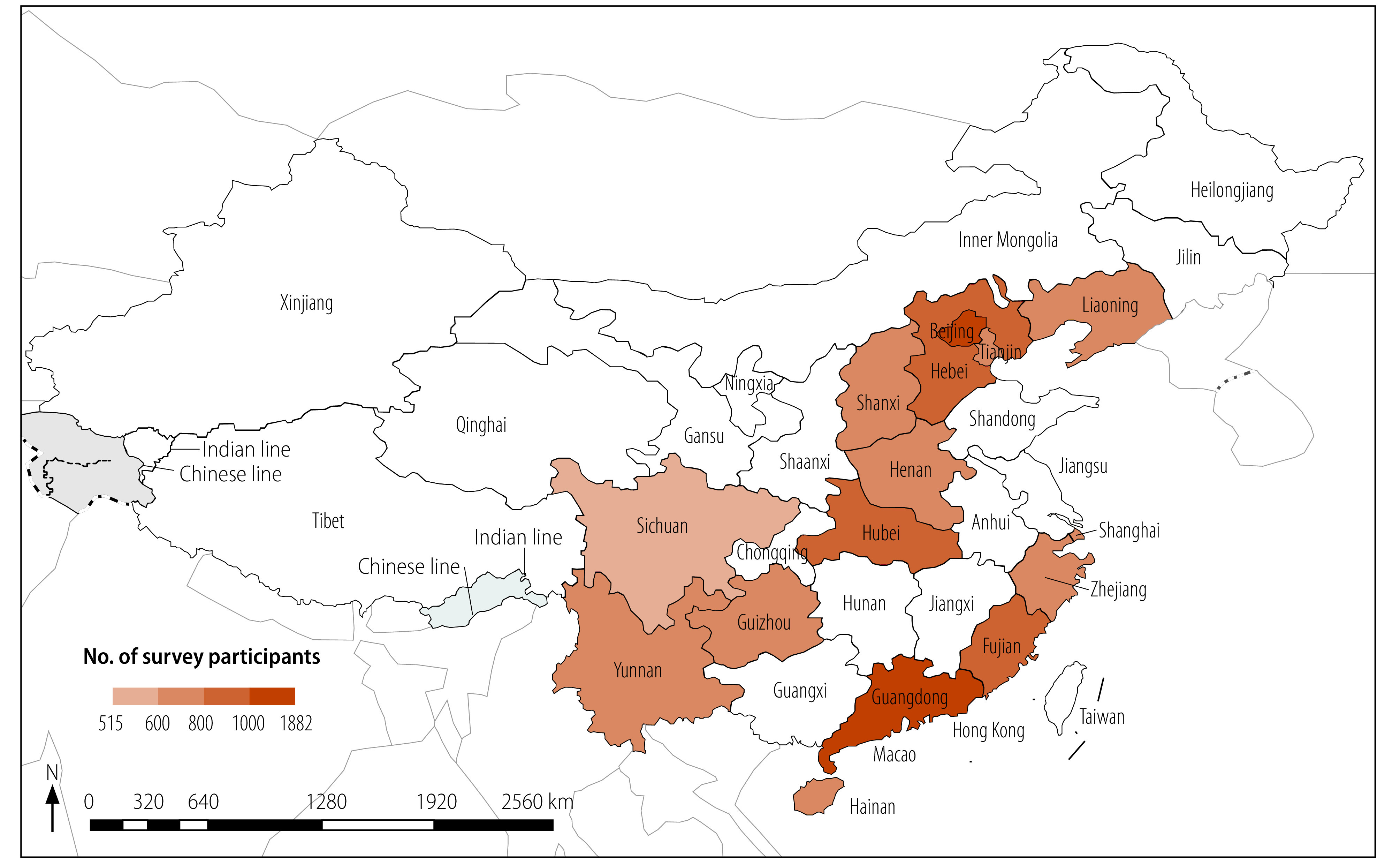
Provinces included in the survey of fertility intention and fecundity in married women, China, 2019–2020

We used a multistage, stratified, sampling method to select a representative sample of women of reproductive age. First, we selected 15 provinces with experience of fertility surveys from the 31 provinces of mainland China (Fig. 1): (i) Fujian, Guangdong, Hainan, Shanghai and Zhejiang in south-east China; (ii) Guizhou, Sichuan and Yunnan in south-west China; (iii) Beijing, Hebei, Henan, Hubei, Shanxi and Tianjin in central China; and (iv) Liaoning in north-east China. We did not include north-west China due to the difficulty of fieldwork in this area. Second, we divided all townships or districts in each province into nine strata according to their degree of urbanization and population size, and selected three townships or districts from each of the highest, middle and lowest strata. Third, we used a random sampling method to select two to four villages or residential areas in each township or district according to their representativeness (i.e. urban or rural location and population size), and to the number of trained interviewers available in each province. In each selected village or residential area, 100 married women aged 20 to 49 years who had been resident for at least six of the previous 12 months before the survey were invited to participate. The sample size met generally recommended requirements for precision in a complex survey design. All participants signed informed consent forms.

Each survey participant completed a questionnaire on: (i) demographic characteristics; (ii) socioeconomic status; (iii) the lifestyle habits of the women and their partners; and (iv) reproductive health. Then they underwent a free medical evaluation, which included: (i) a physical examination; (ii) body mass index assessment; (iii) blood pressure measurement; (iv) a gynaecological examination; (v) a blood test to determine reproductive hormone levels; and (vi) a pelvic ultrasound examination of the uterus and ovaries. Pelvic ultrasound examinations were scheduled to avoid menstrual bleeding.^12^^,^^13^ All study investigators completed a training programme, and qualified clinical staff were trained to obtain blood specimens and perform pelvic ultrasound examinations according to a standard protocol. The survey was approved by the research ethics committees at all participating centres and by the Peking University Third Hospital Ethics Board (protocol 2019SZ-054).

### Outcomes

Fertility intention has previously been defined in several ways, which generally reflect desires, attitudes or behaviours.^15^ We assessed fertility intention by asking the question, “Are you trying to get pregnant now?” Then we classified fertility intention according to whether the woman had the desire or intent to become pregnant and had had unprotected sexual intercourse in the year before the survey visit.

Infertility is defined by the International Committee for Monitoring Assisted Reproductive Technologies and the World Health Organization (WHO) as the failure to achieve a pregnancy after 12 months or more of regular unprotected sexual intercourse.^16^ In the 2020 China Fertility Survey of Married Women, participants who had experienced any 12-month period when they tried to become pregnant but had not conceived, or who took more than 12 months to conceive, were considered infertile, regardless of whether they had children or not. In addition, we also considered participants who underwent assisted reproduction treatment to achieve pregnancy as infertile. Women who did not report having children at the time of survey were defined as childless, regardless of whether they had given birth or not.

Although ovarian reserve has been used as a potential marker of fecundity, the definition of a diminished ovarian reserve remains imprecise, especially for women of childbearing age.^17^^–^^19^ In this epidemiological study, we assessed ovarian reserve by measuring the serum anti-Müllerian hormone level and by counting the number of antral follicles in ovarian tissue during pelvic ultrasonography. An abnormal ovarian reserve was defined as an anti-Müllerian hormone level less than 1.1 ng/mL and an antral follicle count less than seven.^19^

### Statistical analysis

We calculated outcome estimates using sampling weights and post-stratification weights. For each stratum in the sampling process, the sampling weight equalled the reciprocal of the relevant sampling probability. Post-stratification weighting was based on age, with a standardized population derived using data from the sixth national population census in 2010.^20^ Taylor series linearization was used to estimate standard errors and to determine 95% confidence intervals (95% CIs) for prevalence estimates. Odds ratios (ORs) and 95% CIs for fertility intention were estimated for various sociodemographic characteristics using a multivariate logistic regression model. Logistic regression models were also used to estimate age-adjusted ORs for infertility; childlessness; and an abnormal ovarian reserve for various sociodemographic, reproductive and health characteristics. We also conducted a sensitivity analysis among women surveyed before 1 January 2020 to assess the influence of the COVID-19 pandemic. All statistical tests were two-sided and a *P*-value under 0.05 was considered significant. All analyses were performed using R version 3.4.2 (The R Foundation, Vienna, Austria).

## Results

**Fig. 2:**
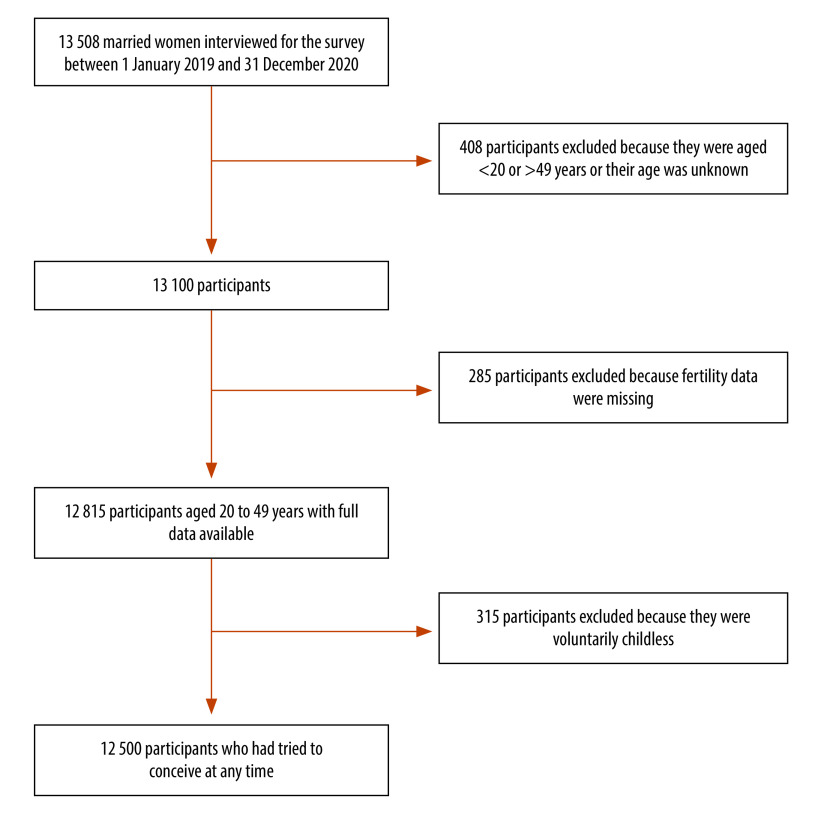
Participant flowchart, survey of fertility intention and fecundity in married women, China, 2019–2020

In total, 13 508 married women were interviewed between January 2019 and December 2020. After excluding 408 women younger than 20 or older than 49 years at the time of the survey visit, and 285 whose fertility condition was unknown (Fig. 2), 12 815 women (mean age: 36.8 years) were included in the analysis (Table 1). As 315 women were voluntarily childless, only 12 500 of the 12 815 participants had previously attempted to conceive.

**Table 1:** Characteristics of participants, survey of fertility intention and fecundity in married women, China, 2019–2020

Survey participants’ characteristic	Value (*n* = 12 815)^a^
**Age in years^b^**	
Mean (SD)	36.8 (7.1)
Median (IQR)	36.7 (31.2–42.8)
Age range, no. (%)	
20–24	518 (4.0)
25–29	1 944 (15.2)
30–34	3 017 (23.5)
35–39	2 731 (21.3)
40–44	2 383 (18.6)
45–49	2 222 (17.3)
**No. of children (%)**	
0	2 290 (17.9)
1	6 451 (50.3)
≥ 2	4 002 (31.2)
Missing data	72 (0.6)
**Household location, no. (%)**	
Urban area	6 884 (53.7)
Rural area	5 821 (45.4)
Missing data	110 (0.9)
Metropolis^c^	
No	9 362 (73.1)
Yes	3 453 (26.9)
**Educational level, no. (%)**	
Primary school	5 449 (43.4)
Higher than primary school	7 223 (55.5)
Missing data	143 (1.1)
**Annual household income in yuan per capita,^d^ no. (%)**	
< 20 000	4 497 (35.0)
20 000 to 50 000	4 549 (35.8)
> 50 000	3 593 (27.8)
Missing data	176 (1.4)
**Body mass index in kg/m^2^**	
Mean (SD)	23.2 (4.1)
Range, no. (%)	
< 18.5	734 (5.7)
18.5–23.9	7 528 (58.7)
24.0–27.9	3 220 (25.1)
≥ 28.0	1 192 (9.3)
Missing data	141 (1.1)
**Current tobacco smoker, no. (%)**	
No	12 170 (95.0)
Yes	548 (4.3)
Missing data	97 (0.8)
**Current alcohol consumption, no. (%)**	
No	10 795 (84.2)
Yes	1 567 (12.2)
Missing data	453 (3.5)

IQR: interquartile range; SD: standard deviation.

^a^ The values listed are for raw data, before sampling and post-stratification weighting.

^b^ All women surveyed were aged between 20 and 49 years.

^c^ The metropolises included in the survey were Beijing, Guangzhou, Shanghai and Shenzhen.

^d^ During 2019 and 2020, 1 yuan was equivalent to 0.14 United States dollars on average.

After applying weighting, we estimated that 11.9% of women reported trying to become pregnant at the time of the survey visit (Table 2). There was a significant decline in fertility intention with advancing age (*P* < 0.05), with 9.3% of 2731 women aged 35 to 39 years still trying to become pregnant. Among 2290 childless women, 25.4% were trying to become pregnant at the time of the visit. In contrast, of the 10 453 women reporting having one or more children, only 6.1% wanted to conceive another. Fertility intention was significantly lower among the 3453 women residing in a metropolis than among the 9362 not in a metropolis (OR: 0.38; 95% CI: 0.31–0.45); and among the 7223 women with an education higher than primary school than in the 5449 with primary education only (OR: 0.74; 95% CI: 0.62–0.88). In addition, women with a higher body mass index were more likely to report fertility intention (*P* < 0.05). Findings were similar in a sensitivity analysis conducted to investigate the impact of the COVID-19 pandemic on fertility intentions, which included 11 937 women surveyed before 1 January 2020.

**Table 2:** Factors associated with fertility intention, survey of fertility intention and fecundity in married women, China, 2019–2020

Survey participants’ characteristic	% of participants trying to conceive (95% CI)^a^	Likelihood of trying to conceive, OR (95% CI)^b^
**All participants**	11.9 (10.9–13.0)	NA
**Age, years**		
20–24	18.5 (15.0–22.8)	Reference
25–29	18.7 (16.7–20.8)	1.54 (1.15–2.06)
30–34	14.5 (13.1–16.1)	2.61 (1.94–3.53)
35–39	9.3 (8.1–10.7)	2.51 (1.82–3.48)
40–44	5.2 (4.2–6.4)	1.11 (0.77–1.59)
45–49	1.5 (1.0–2.4)	0.30 (0.18–0.48)
**No. of children**		
0	25.4 (22.7–28.3)	Reference
≥ 1	6.1 (5.4–6.8)	0.02 (0.02–0.03)
**Household location**		
Urban area	9.9 (8.9–11.1)	Reference
Rural area	13.8 (12.2–15.6)	1.30 (1.12–1.53)
Metropolis^c^		
No	13.2 (12.1–14.4)	Reference
Yes	6.4 (4.8–8.3)	0.38 (0.31–0.45)
**Educational level**		
Primary school	11.8 (10.1–13.7)	Reference
Higher than primary school	12.2 (11.1–13.5)	0.74 (0.62–0.88)
**Annual household income, yuan per capita^d^**		
< 20 000	12.5 (10.8–14.4)	0.99 (0.84–1.17)
20 000–50 000	12.8 (11.1–14.7)	Reference
> 50 000	10.3 (8.9–12.0)	0.86 (0.72–1.02)
**Current tobacco smoker**		
No	11.9 (10.9–13.0)	Reference
Yes	12.8 (8.9–18.1)	1.29 (0.96–1.72)
**Current alcohol consumption**		
No	12.2 (11.1–13.4)	Reference
Yes	10.9 (8.5–13.9)	0.77 (0.62–0.94)
**Body mass index, kg/m^2^**		
< 18.5	13.0 (9.6–17.5)	0.86 (0.66–1.13)
18.5–23.9	11.0 (9.9–12.3)	Reference
24.0–27.9	10.9 (9.1–12.9)	1.21 (1.02–1.43)
≥ 28.0	19.1 (14.8–24.4)	1.64 (1.31–2.04)

CI: confidence interval; NA: not applicable; OR: odds ratio.

^a^ The figures listed are for weighted proportions based on sampling weights and post-stratification weights.

^b^ Odds ratio were estimated using a multivariate logistic regression model that included all covariables listed in the table.

^c^ The metropolises included in the survey were Beijing, Guangzhou, Shanghai and Shenzhen.

^d^ During 2019 and 2020, 1 yuan was equivalent to 0.14 United States dollars on average.

Table 3 shows the estimated odds of infertility and childlessness among the 12 500 women who tried to become pregnant, according to sociodemographic characteristics and ovarian reserve. Of these women, 2237 reported they had unsuccessfully tried to conceive spontaneously for 12 months or more in the past. The weighted prevalence of infertility was 18.0% (95% CI: 16.9–19.0). As indicated by age-adjusted odds ratios, the prevalence of infertility was significantly lower among women who were living in a metropolis (*P* < 0.05), and tended to be lower in those who had a high annual household income or a high educational level. In contrast, the prevalence of infertility was higher among obese women (*P* < 0.05), defined as those with a body mass index of 28 kg/m^2^ or more.

**Table 3:** Factors associated with infertility and childlessness, survey of fertility intention and fecundity in married women, China, 2019–2020

Survey participants’ characteristic	Infertility^a ^(*n* = 12 500)			Childlessness (*n* = 12 500)	
	% of participants (95% CI)^b^	Age-adjusted OR (95% CI)^c^		% of participants (95% CI)^b^	Age-adjusted OR (95% CI)^c^
**All participants**	18.0 (16.9–19.0)	NA		29.1 (27.4–31.0)	NA
**Age, years**					
20–24	16.3 (12.6–20.9)	Reference		73.6 (68.4–78.1)	Reference
25–29	20.4 (18.3–22.7)	1.31 (0.94–1.82)		44.6 (41.9–47.3)	0.29 (0.22–0.38)
30–34	21.1 (19.4–22.9)	1.37 (1.00–1.88)		18.7 (17.0–20.5)	0.08 (0.06–0.11)
35–39	18.7 (17.0–20.6)	1.18 (0.86–1.63)		7.1 (6.1–8.3)	0.03 (0.02–0.04)
40–44	16.5 (14.8–18.3)	1.01 (0.73–1.4)		4.6 (3.7–5.6)	0.02 (0.01–0.02)
45–49	15.3 (13.5–17.2)	0.92 (0.66–1.29)		5.2 (4.2–6.5)	0.02 (0.01–0.03)
**Household location**					
Urban area	18.2 (16.9–19.5)	Reference		32.5 (29.9–35.2)	Reference
Rural area	17.6 (15.9–19.5)	0.94 (0.82–1.09)		25.5 (23.5–27.6)	0.71 (0.59–0.85)
Metropolis^d^					
No	19.2 (18.0–20.6)	Reference		29.4 (27.6–31.3)	Reference
Yes	12.6 (10.7–14.8)	0.60 (0.49–0.74)		27.7 (24.0–31.6)	0.76 (0.60–1.00)
**Educational level**					
Primary school	18.6 (16.8–20.6)	Reference		19.0 (16.6–21.6)	Reference
Higher than primary school	17.7 (16.4–19.1)	0.91 (0.77–1.09)		36.4 (34.2–38.7)	2.37 (1.92–2.91)
**Annual household income, yuan per capita^e^**					
< 20 000	19.3 (17.3–21.4)	Reference		30.8 (27.9–33.9)	Reference
20 000–50 000	17.6 (15.8–19.6)	0.91 (0.75–1.09)		28.0 (25.3–30.9)	1.10 (0.89–1.36)
> 50 000	17.3 (15.4–19.3)	0.88 (0.73–1.06)		27.4 (24.5–30.4)	1.14 (0.91–1.43)
**Current tobacco smoker**					
No	17.9 (16.8–19.1)	Reference		28.4 (26.7–30.1)	Reference
Yes	18.3 (13.4–24.6)	1.01 (0.68–1.48)		42.1 (33.2–51.5)	1.25 (0.77–2.03)
**Current alcohol consumption**					
No	17.8 (16.6–19.1)	Reference		27.3 (25.5–29.1)	Reference
Yes	17.6 (14.4–21.2)	0.96 (0.75–1.23)		39.2 (34.1–44.6)	1.28 (0.99–1.66)
**Body mass index, kg/m^2^**					
< 18.5	17.3 (13.1–22.5)	1.02 (0.72–1.44)		55.1 (48.7–61.3)	1.70 (1.28–2.26)
18.5–23.9	16.5 (15.2–17.9)	Reference		28.8 (26.7–31.1)	Reference
24.0–27.9	18.1 (16.1–20.3)	1.14 (0.96–1.36)		20.1 (17.3–23.2)	0.97 (0.78–1.21)
≥ 28.0	27.1 (22.2–32.7)	1.89 (1.43–2.50)		28.0 (22.8–33.8)	0.93 (0.65–1.33)
**Abnormal ovarian reserve^f^**					
No	18.0 (16.8–19.3)	Reference		32.6 (30.7–34.5)	Reference
Yes	17.5 (15.4–19.8)	1.05 (0.86–1.28)		7.0 (5.5–9.0)	1.78 (1.36–2.33)

CI: confidence interval; NA: not applicable; OR: odds ratio.

^a^ Infertility included primary and secondary infertility.

^b^ The figures listed are for weighted proportions based on sampling weights and post-stratification weights.

^c^ We estimated ORs using a logistic regression model that included adjustment for age.

^d^ The metropolises included in the survey were Beijing, Guangzhou, Shanghai and Shenzhen.

^e^ During 2019 and 2020, 1 yuan was equivalent to 0.14 United States dollars on average.

^f^ We defined an abnormal ovarian reserve as an anti-Müllerian hormone level less than 1.1 ng/mL and an antral follicle count under seven.

Overall, 2209 of the 12 500 women who had previously attempted to conceive were childless at the time of the survey (weighted prevalence: 29.1%; 95% CI: 27.0–30.0; Table 3). The prevalence of childlessness declined significantly with increasing age (*P* < 0.05): it was 7.1% in women aged 35 to 39 years, compared with 4.6% in those aged 40 to 44 yearsy and 5.2% in those aged 45 to 49 years. As indicated by age-adjusted odds ratios, the proportion of women who were childless was significantly higher among urban women than among those living in rural locations, among women with a higher educational level and among women with a body mass index of 18.5 kg/m^2^ or less (*P* < 0.05 for all). In addition, women with an abnormal ovarian reserve had a significantly elevated risk of being childless (*P* < 0.05).

Table 4 shows the estimated odds of an abnormal ovarian reserve among the women surveyed, according to sociodemographic characteristics. Of the 10 155 participants who underwent a blood test, 3382 had an anti-Müllerian hormone level under 1.1 ng/mL (weighted prevalence: 30.4%; 95% CI: 28.9–32.0). In addition, of the 11 710 participants who underwent pelvic ultrasound examination, 3551 had an antral follicle count less than seven (weighted prevalence: 25.8%; 95% CI: 24.7–27.0). We observed that the ovarian reserve declined significantly with increasing age (*P* < 0.05). In particular, the prevalence of an abnormal ovarian reserve rose sharply after the age of 35 years. As indicated by age-adjusted odds ratios, the proportion of women with an abnormal anti-Müllerian hormone level was significantly lower in those who lived in a metropolis, had a higher educational level or had a higher annual household income (*P* < 0.05 for all). However, no significant association was found between the antral follicle count and any socioeconomic characteristic. The prevalence of an abnormal ovarian reserve was significantly higher in obese women (*P* < 0.05).

**Table 4:** Factors associated with an abnormal ovarian reserve, survey of fertility intention and fecundity in married women, China, 2019–2020

Survey participants’ characteristic	Abnormal ovarian reserve				
	Anti-Müllerian hormone level < 1.1 ng/mL (*n* = 10 155)^a^			Antral follicle count < 7 (*n* = 11 710)^b^	
	% of participants^c^ (95% CI)	Age-adjusted OR (95% CI)^d^		% of participants^c^ (95% CI)	Age-adjusted OR (95% CI)^d^
**All participants**	30.4 (28.9–32.0)	NA		25.8 (24.7–27.0)	NA
**Age, years**					
20–24	5.0 (2.5–9.5)	Reference		4.0 (2.5–6.4)	Reference
25–29	6.5 (4.9–8.5)	1.32 (0.62–2.83)		4.1 (3.2–5.3)	1.01 (0.58–1.74)
30–34	11.5 (9.7–13.5)	2.48 (1.20–5.11)		8.6 (7.5–9.9)	2.24 (1.36–3.69)
35–39	26.1 (23.3–29)	6.77 (3.32–13.8)		22.3 (20.5–24.3)	6.82 (4.18–11.1)
40–44	61.3 (58.2–64.3)	30.4 (14.9–61.8)		50.1 (47.5–52.7)	23.8 (14.6–38.8)
45–49	90.2 (88.2–92.0)	177 (85.3–367)		76.7 (73.9–79.4)	78.2 (47.4–129)
**Household location**					
Urban area	33.7 (31.6–35.8)	Reference		27.8 (26.4–29.3)	Reference
Rural area	27.0 (25.0–29.2)	1.03 (0.86–1.23)		23.9 (22.4–25.4)	1.02 (0.89–1.16)
Metropolis^e^					
No	31.2 (29.5–33.0)	Reference		25.3 (24.2–26.5)	Reference
Yes	26.8 (23.9–30.0)	0.77 (0.65–0.93)		27.6 (25.2–30.1)	1.12 (0.98–1.29)
**Educational level**					
Primary school	41.6 (38.9–44.3)	Reference		34.5 (32.6–36.4)	Reference
Higher than primary school	23.0 (21.3–24.7)	0.79 (0.63–1.00)		19.5 (18.3–20.7)	0.93 (0.8–1.09)
**Annual household income, yuan per capita^e^**					
< 20 000	32.0 (29.4–34.7)	Reference		24.9 (23.1–26.7)	Reference
20 000–50 000	32.2 (29.8–34.7)	0.80 (0.64–1.00)		29.2 (27.4–31.1)	1.10 (0.94–1.29)
> 50 000	25.9 (23.2–28.8)	0.68 (0.54–0.85)		22.5 (20.6–24.4)	0.95 (0.8–1.13)
**Current tobacco smoker**					
No	30.7 (29.2–32.3)	Reference		26.1 (25.0–27.2)	Reference
Yes	23.2 (16.9–30.9)	0.91 (0.63–1.30)		18.2 (14.1–23.1)	0.92 (0.67–1.28)
**Current alcohol consumption**					
No	31.8 (30.1–33.5)	Reference		27.2 (26.1–28.4)	Reference
Yes	21.9 (18.5–25.8)	0.83 (0.65–1.06)		17.0 (14.7–19.5)	0.76 (0.62–0.93)
**Body mass index, kg/m^2^**					
< 18.5	10.9 (8.2–14.3)	0.82 (0.55–1.21)		10.5 (8.1–13.4)	0.97 (0.70–1.35)
18.5–23.9	28.5 (26.6–30.5)	Reference		24.0 (22.7–25.4)	Reference
24.0–27.9	40.0 (36.8–43.2)	1.09 (0.92–1.30)		34.1 (31.8–36.5)	0.97 (0.84–1.11)
≥ 28.0	35.5 (30.2–41.1)	1.30 (1.00–1.70)		30.8 (26.9–35.1)	1.35 (1.03–1.77)
**Current fertility intention^f^**					
No	33.0 (31.4–34.7)	Reference		27.7 (26.5–28.9)	Reference
Yes	13.9 (11.2–17.1)	0.98 (0.71–1.37)		12.2 (9.9–14.9)	1.16 (0.87–1.54)

CI: confidence interval; NA: not applicable; OR: odds ratio.

^a^ Of the 12 815 survey participants, 10 155 had a blood test for reproductive hormones.

^b^ Of the 12 815 survey participants, 11 710 underwent a pelvic ultrasound examination.

^c^ The figures listed are for weighted proportions based on sampling weights and post-stratification weights.

^d^ We estimated odds ratio using a logistic regression model that included adjustment for age.

^e^ The metropolises included in the survey were Beijing, Guangzhou, Shanghai and Shenzhen.

^f^ Fertility intention was the desire or intent to become pregnant combined with engagement in unprotected sexual intercourse.

## Discussion

In this representative sample of married Chinese women of reproductive age, 11.9% overall reported fertility intentions. The proportion was only 6.1% in women who had already given birth to one or more children. In addition, 18.0% had experienced infertility and almost 30% had an abnormal ovarian reserve at the time of the survey. These findings can help guide a re-examination of population policy in China.

Almost all previously available data on fertility intentions have been based on demographic indicators, such as the ideal number of children or intended family size,^15^ which has limited our understanding of desired fertility. Our study, which used individual epidemiological data, found that 25.4% of women who had no children were trying to conceive compared with 6.1% of women who already had children. A 2011 study revealed that 74% of childless women aged 20 to 40 years in France and 85% of similar women in Italy intended to have a child. Moreover, 62% of mothers with one child in France and 53% in Italy intended to have a second child.^21^ In addition, a survey conducted in Australia in 2011 estimated that 21.8% of women aged 18 to 41 years had a strong desire to conceive.^22^ Although the age compositions of the women sampled in these studies differ from those in our survey, the numbers indicate that, even with a relaxed population policy, the proportion of Chinese women of childbearing age who want to bear children is low.

Generally, the predominant reason for a low fertility rate is a decline in fertility intentions. In China, the traditional division of labour in families persists: men typically serve as breadwinners, while women assume the role of primary caregivers. Under the system of public ownership, work–family conflicts were relatively well reconciled for urban working women.^23^ However, with economic globalization, an increasing number of women in recent decades have prioritized their education and career, which has resulted in a widespread trend towards delayed motherhood.^24^ Moreover, faced with a scarcity of childcare provision and the increasing cost of raising children (e.g. investment in education) during the transition to marketization,^23^ a substantial proportion of young citizens decided to postpone becoming parents, or to opt for fewer children, or both. These factors could explain the low fertility intentions among women with a high socioeconomic status that we observed.

Poor fecundity is another reason for low fertility. In our study, we assessed the fecundity of Chinese women of reproductive age using biological markers and found that almost a third had an abnormal ovarian reserve. A low anti-Müllerian hormone level appears to be correlated with a low number of antral follicles and is indicative of a low ovarian reserve, which is, in turn, associated with poor fertility.^19^ The anti-Müllerian hormone level and the antral follicle count are less influenced by the menstrual cycle than levels of other reproductive hormones related to fertility. Consequently, in large-scale epidemiological surveys, these two markers can give a better indication of the potential fertility of the general population.

Although the reasons for the general reduction in ovarian reserve are not fully understood, it is widely accepted that the life stress and environmental pollution accompanying industrialization could be associated with premature ovarian aging.^19^ One possible result of an accelerated general decline in ovarian reserve is that a woman who has an abnormal ovarian reserve at an early age could have an increased risk of infertility, pregnancy loss and even adverse birth outcome.^25^ Combined with the trend of delayed motherhood, these changes in ovarian reserve suggest that a large well-educated section of the Chinese population may be affected by infertility, have poor responses to fertility treatment and will even remain childless in the future. 

In a previous study in 2010, we found that the prevalence of infertility in China was 15.5%.^12^ Following the easing of the one-child policy in 2015, some older mothers attempted to have a second child, which may have increased the recorded prevalence of infertility. In the present study, we reported a prevalence of 18.0% after adjusting for sampling weights and the age composition of the population. This rate was slightly higher than that observed in developed countries like Finland (16.0% in 2002) and Norway (12.7% in 2006),^26^^,^^27^ as well as in China a decade ago.^12^ More worrying is the number of women who remain unable to conceive.^5^ In 2020, 56% of infertile couples sought fertility treatment in China.^28^ However, the live birth rate with assisted reproductive technologies hovers around 25% in China, Europe and the United States of America.^28^ Consequently, not only can it be expensive to treat infertility, but the resulting unintentional childlessness imposes a substantial burden on both individuals and society.

In our study, less than one fifth of married Chinese women under 30 years of age expressed fertility intentions at the time of the survey. Moreover, fertility intention was even less common among women living in a metropolis and in those with a higher educational level. The difficulty of combining motherhood and employment appears to be responsible for delaying, or giving up, having a child. On the other hand, although women with a relatively low socioeconomic status were more likely to have fertility intentions, they were also more likely to have an abnormal ovarian reserve. Hence, the cause of low fertility can differ among various demographic groups. For newly industrialized countries with low fertility rates, below-replacement fertility, rapidly aging populations and looming labour shortages, it is critically important that policies are introduced to improve women’s reproductive health and the living standards of families with children. Infertility and unintended childlessness in women of childbearing age could be alleviated by monitoring ovarian reserves and devising a childbirth plan.

In our study, we also observed that obesity was associated with fertility intention, infertility and an abnormal ovarian reserve. Evidence suggests that being obese can lead to a decrease in the number and volume of oocytes, thereby inducing hormonal dysfunction and, potentially, irregular ovulation or even anovulation.^29^ A recent study of 2.3 million Chinese couples who wanted to conceive found that the pregnancy rate was 19% lower in obese women than in those with a normal body mass index.^30^ Obesity can be a manifestation of the metabolic complications of polycystic ovary syndrome, and there is an indication that obese patients with the syndrome have a more severe phenotype than those with a normal weight.^31^ Overweight and obesity are increasingly prevalent and have extensive adverse consequences.^32^ Consequently, lifestyle interventions to achieve a healthy weight are of great importance, both in themselves and for intergenerational health.

Our study had several limitations. First, information on reproductive history was collected retrospectively in the survey. Even though we designed a series of logically related questions to determine reproductive history, and excluded questionnaires with logical errors from our analysis, the estimated infertility rate may have been influenced by recall bias, particularly when the follow-up time was long.^33^ Second, in the villages and streets selected, the response rate among young women may have been low because many were studying or working in another city. Consequently, potential selection errors should be considered when interpreting our prevalence rates. Nevertheless, these limitations were unlikely to have distorted our results because our sample size was large and we calculated age-adjusted rates.

Our definitions of fertility intention and infertility should be noted. Fertility intention at the time of survey was conceptualized as the desire or intent to become pregnant combined with a reported engagement in unprotected sexual intercourse. This definition may have resulted in an underestimate of fertility intention compared with that derived from demographic indicators, such as the ideal number of children and intended family size. 

Furthermore, it is noteworthy that participants were visited by medical personnel between January 2019 and December 2020. The outbreak of the global COVID-19 pandemic was officially declared by WHO on 11 March 2020. As the global pandemic had the potential to influence fertility intentions, we conducted a sensitivity analysis in which we excluded 878 participants who were visited after 1 January 2020. We found that our conclusions were unlikely to have been substantially altered by the pandemic.

Finally, unlike studies that investigated infertility at a specific point in time (e.g. in the preceding year), which can minimize potential recall bias in reproductive epidemiology studies,^8^ we used infertility at any point in a woman’s lifetime to estimate the overall burden of infertility among women of childbearing age.

In conclusion, the willingness of Chinese married women to give birth continues to be low despite the government’s relaxation of the one-child policy in 2013. Better targeted population stimulus policies may be needed to boost China’s low fertility rate.
